# Efficacy and safety of mono antiplatelet therapy with colchicine in acute coronary syndrome patients following percutaneous coronary intervention: rationale and design of the MACT II trial

**DOI:** 10.3389/fcvm.2025.1662392

**Published:** 2025-10-27

**Authors:** Ji Yong Jang, Yongsung Suh, Choongki Kim, Jeong Tae Byoun, Kyeong Ho Yun, Jung-Hee Lee, Ki-Hyun Jeon, Sungsoo Cho, Hyuk-Joon Yoon, Jin Won Kim, Bom Lee, Se Hun Kang, Sang-Hoon Kim, Jae Youn Moon, Yangsoo Jang, Seung-Yul Lee

**Affiliations:** ^1^ National Health Insurance Service Ilsan Hospital, Goyang, Republic of Korea; ^2^ Cardiovascular Center, Myongji Hospital, Goyang, Republic of Korea; ^3^ Ewha Womans University Seoul Hospital, Seoul, Republic of Korea; ^4^ Regional Cardiocerebrovascular Center, Wonkwang University Hospital, Ikstan, Republic of Korea; ^5^ Wonju Severance Christian Hospital, Wonju, Republic of Korea; ^6^ Seoul National University Bundang Hospital, Seongnam, Republic of Korea; ^7^ Gangnam Severance Hospital, Seoul, Republic of Korea; ^8^ Kyemyung University Dongsan Hospital, Daegu, Republic of Korea; ^9^ Korea University Guro Hospital, Seoul, Republic of Korea; ^10^ CHA Bundang Medical Center, Seongnam, Republic of Korea; ^11^ Health Insurance Review and Assessment Service, Wonju, Republic of Korea

**Keywords:** acute coronary syndrome, percutaneous coronary intervention, aspirin, colchicine, ticagrelor

## Abstract

**Introduction:**

Early discontinuation of aspirin after percutaneous coronary intervention (PCI) reduces bleeding risk, while inflammation-targeted strategies may offer additional benefit in patients with acute coronary syndrome (ACS). Residual inflammatory risk—reflected by persistently elevated high-sensitivity C-reactive protein (hs-CRP) levels—remains a significant contributor to adverse cardiovascular outcomes despite guideline-directed therapy. Colchicine has emerged as a potential anti-inflammatory agent in this context, but its optimal use remains uncertain.

**Methods and analysis:**

MACT (Mono Antiplatelet and Colchicine Therapy) II is an investigator-initiated, prospective, multicenter, single-arm study designed to evaluate the safety and efficacy of an aspirin-free, inflammation-guided treatment strategy in 490 patients with troponin-positive ACS or ST-segment elevation myocardial infarction undergoing PCI with a sirolimus-eluting stent. Aspirin is discontinued on the day after PCI, and ticagrelor is continued at the standard dose. Colchicine (0.6 mg once daily) is initiated within 24 h after PCI. At 1 month, colchicine is continued or discontinued based on hs-CRP levels. The primary outcome is the 12-month incidence of net adverse clinical events, a composite of cardiovascular death, nonfatal myocardial infarction, nonfatal ischemic stroke, unplanned urgent revascularization, and major bleeding (Bleeding Academic Research Consortium type 3 or 5). Secondary outcomes include longitudinal hs-CRP trends, platelet reactivity, and adverse drug reactions.

**Conclusions:**

MACT II evaluates an aspirin-free, inflammation-guided treatment strategy in patients with ACS undergoing PCI. Colchicine therapy is initiated early during the acute phase of ACS and continued or discontinued based on inflammatory response as measured by hs-CRP. By tailoring treatment duration in this manner, the trial aims to reduce both ischemic and bleeding risks while avoiding unnecessary drug exposure. The findings may inform personalized anti-inflammatory strategies in contemporary clinical practice.

**Trial registration:**

ClinicalTrials.gov Identifier: NCT06543082.

## Introduction

Acute coronary syndrome (ACS) remains a leading cause of morbidity and mortality worldwide. The cornerstone of treatment following percutaneous coronary intervention (PCI) is dual antiplatelet therapy (DAPT), typically combining aspirin with a P2Y12 inhibitor to reduce thrombotic events (1, 2). However, prolonged DAPT significantly increases bleeding risks, highlighting the need for strategies that maintain antithrombotic efficacy while minimizing bleeding complications. Recent trials, including TWILIGHT, TICO, and ULTIMATE-DAPT (3–5), demonstrated that transitioning from DAPT to P2Y12 inhibitor monotherapy after a brief period effectively reduces bleeding without compromising ischemic protection. Despite this progress, patients remain at substantial risk for recurrent cardiovascular events, underscoring the need to address factors beyond platelet activation—particularly residual inflammation, which critically contributes to long-term cardiovascular risk.

Inflammation plays a central role in ACS pathogenesis, driving plaque rupture, thrombus formation, and subsequent ischemic complications. Persistently elevated high-sensitivity C-reactive protein (hs-CRP), a marker of systemic inflammation, indicates ongoing inflammatory risk even after clinical stabilization (6). Residual inflammatory risk, reflected by persistently elevated hs-CRP (≥2 mg/L) after PCI, independently predicts recurrent cardiovascular events such as myocardial infarction and stroke (7, 8). Consequently, targeting residual inflammation may offer an additional therapeutic benefit when layered onto modern antiplatelet strategies. Colchicine, a potent anti-inflammatory agent, has emerged as an effective option to reduce residual inflammation and improve cardiovascular outcomes. Landmark studies such as COLCOT and LoDoCo2 have shown that colchicine significantly reduces recurrent events in patients with recent myocardial infarction or stable coronary syndromes (9, 10). Moreover, substituting colchicine for aspirin may further lower bleeding risk, potentially offering a safer and more effective long-term treatment strategy following PCI.

Building upon these concepts, the MACT pilot study evaluated the feasibility of replacing aspirin with colchicine, combined with ticagrelor or prasugrel monotherapy, in ACS patients after PCI (11). In that study, a fixed 3-month colchicine regimen was well tolerated, maintained effective platelet inhibition, and significantly reduced residual inflammation, suggesting clinical feasibility for a colchicine-based, aspirin-free approach (11). The MACT II trial was designed to further investigate this approach in a larger, multicenter setting. A key feature of the study is the use of hs-CRP-guided colchicine therapy, in which continuation of treatment is based on the persistence of systemic inflammation (hs-CRP ≥2 mg/L) at 1 month post-PCI. This strategy enables individualized, inflammation-guided therapy that may optimize risk-benefit balance by selectively extending treatment in patients with ongoing inflammatory activity.

We hypothesize that an hs-CRP–guided, aspirin-free strategy combining ticagrelor monotherapy and colchicine will effectively reduce both ischemic and bleeding events in ACS patients undergoing PCI. The MACT II trial aims to evaluate the efficacy and safety of this personalized anti-inflammatory approach, with serial hs-CRP measurements during follow-up providing insights into the inflammatory response.

## Methods and analysis

### Study design

**Figure 1:**
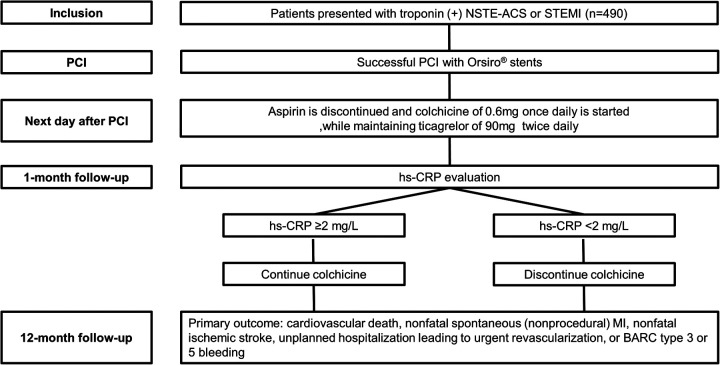
Study flow diagram. BARC, bleeding academic research consortium; hs-CRP, high-sensitivity C-reactive protein; MI, myocardial infarction; NSTE-ACS, non-ST-elevation acute coronary syndrome; PCI, percutaneous coronary intervention; STEMI, ST-segment elevation myocardial infarction.

The MACT II trial is an investigator-initiated, prospective, multicenter, single-arm clinical trial designed to evaluate the efficacy and safety of ticagrelor monotherapy combined with low-dose colchicine in ACS patients undergoing PCI. Patients diagnosed with troponin-positive non-ST-elevation ACS or ST-segment elevation myocardial infarction (STEMI) undergoing PCI with ultrathin bioresorbable polymer sirolimus-eluting stents (Orsiro; Biotronik AG) are eligible for enrollment. After obtaining informed consent and confirming eligibility, aspirin therapy is discontinued the day after PCI, while ticagrelor (90 mg twice daily) is continued. Simultaneously, low-dose colchicine (0.6 mg daily) is initiated. Colchicine treatment duration is guided by hs-CRP measurements at one month after PCI: patients with hs-CRP ≥ 2 mg/L continue colchicine therapy for the study duration, while those with hs-CRP <2 mg/L discontinue colchicine at one month. A schematic overview illustrating patient flow, including enrollment, interventions, and follow-up timelines, is provided in Figure 1.

### Study population

The study population includes patients presenting with ACS, defined as troponin-positive unstable angina/non-ST-elevation myocardial infarction (NSTEMI), or STEMI, who undergo successful PCI with ultrathin bioresorbable polymer sirolimus-eluting stents (Orsiro; Biotronik AG). Patients with cardiac arrest or cardiogenic shock, those requiring anticoagulation therapy, or lesions associated with stent failure (stent restenosis or thrombosis) are excluded. The trial aims to enroll a total of 490 patients, who must meet all eligibility criteria, detailed in Table 1.

**Table 1:** Eligibility criteria.

Inclusion criteria	Exclusion criteria
Patients aged ≥19 years	Age <19 years
Diagnosis of troponin-positive acute coronary syndrome or ST-segment elevation myocardial infarction	Cardiac arrest or cardiogenic shock
Successful percutaneous coronary intervention with ultrathin bioresorbable polymer sirolimus-eluting stents (Orsiro; Biotronik AG)	Requirement for anticoagulation therapy
Provision of signed informed consent	Lesions with stent failure (stent restenosis or thrombosis)
	Renal impairment (creatinine clearance <50 mL/min)
	Severe liver disease (Child-Pugh class B or C)
	Concomitant use or requirement of strong CYP3A4 inhibitors or P-glycoprotein inhibitors
	Hematologic abnormalities: myelosuppression, leukopenia, granulocytopenia, thrombocytopenia, pancytopenia, aplastic anemia
	Severe gastrointestinal disorders
	Pregnant, lactating, or women of childbearing potential not using contraception
	Current malignancy or malignancy within the past 5 years
	Life expectancy <5 years
	Known hypersensitivity or contraindication to colchicine or ticagrelor

### Study procedure

Following successful PCI with ultrathin bioresorbable polymer sirolimus-eluting stents (Orsiro; Biotronik AG), eligible patients are enrolled after providing written informed consent. Aspirin is discontinued on the day after PCI, and ticagrelor (90 mg twice daily) is continued as the maintenance antiplatelet agent. For patients who received a different P2Y12 inhibitor (e.g., clopidogrel) prior to PCI, a loading dose of ticagrelor (180 mg) is administered on the day after PCI, followed by the standard maintenance dose (12).

Colchicine (0.6 mg once daily) is initiated within 24 h after PCI. At one month, hs-CRP levels are measured using a standardized, high-sensitivity immunoturbidimetric assay to guide treatment duration. Patients with hs-CRP ≥ 2 mg/L continue colchicine therapy until 12 months, while those with hs-CRP < 2 mg/L discontinue colchicine at one month.

Clinical follow-up visits are scheduled at 1, 3, 6, 9, and 12 months post-PCI. At each visit, data are collected on symptoms, adverse events, medication adherence, and concomitant medications. Laboratory tests—including hematologic, renal and liver function, lipid profile, cardiac biomarkers, and hs-CRP—are performed at baseline and at 1, 6, and 12 months post-PCI. Platelet function testing using VerifyNow or Thromboelastography is recommended at 1 and 12 months.

### Study outcomes

The primary efficacy outcome is net adverse clinical events, defined as a composite of cardiovascular death, nonfatal spontaneous myocardial infarction, nonfatal ischemic stroke, unplanned hospitalization requiring urgent revascularization, and major bleeding, assessed at 12 months post-PCI. The primary safety outcome is the incidence of stent thrombosis within 12 months, classified as definite, probable, or possible according to the Academic Research Consortium definitions (13).

Cardiovascular death includes fatal events of cardiac or vascular origin, as well as unwitnessed or unknown-cause deaths (13). Spontaneous myocardial infarction is defined by a rise and/or fall in cardiac troponin, with at least one value above the 99th percentile upper reference limit, accompanied by evidence of ischemia (symptoms, ECG changes, or imaging findings) (14). Ischemic stroke is defined as a new focal neurological deficit lasting ≥24 h with imaging confirmation (13). Urgent revascularization refers to unplanned PCI or coronary artery bypass surgery performed during hospitalization for recurrent or worsening ischemic symptoms (15). Major bleeding is defined as type 3 or 5 according to the Bleeding Academic Research Consortium criteria, including bleeding requiring transfusion, surgical intervention, or resulting in death (16).

Secondary outcomes include each component of the primary outcome, as well as measures of inflammatory status, platelet reactivity, thrombogenicity, and drug safety over 12 months post-PCI. High residual inflammation is assessed by the proportion of patients with hs-CRP ≥2 mg/L at 1, 6, and 12 months (7), along with changes in hs-CRP following colchicine discontinuation. High and low residual platelet reactivity, defined as platelet reaction units >208 and <85 respectively (17), and thrombogenicity are evaluated at 1 and 12 months (18). Adverse drug reactions to colchicine are assessed at 1, 3, 6, 9, and 12 months.

All clinical outcomes are adjudicated by an independent Clinical Events Committee blinded to treatment exposure and hs-CRP status. In addition, safety monitoring is conducted by an independent Data and Safety Monitoring Board, which regularly reviews adverse events and overall study conduct to ensure participant safety and study integrity.

### Sample size calculation

The sample size for the MACT II trial was determined based on the expected incidence of net adverse clinical events at 12 months post-PCI. Based on previous data from the TICO trial, the event rate in a comparable population receiving standard therapy was estimated at 8.7% (4). Informed by findings from the MACT pilot study (11), the anticipated event rate in the investigational group—ticagrelor monotherapy combined with colchicine—was projected to be 4.0%. To detect this difference with 80% power and a one-sided alpha of 2.5%, a total of 419 patients were required. Allowing for an estimated 15% loss to follow-up, the final target sample size was set at 490 patients.

### Statistical analysis

The cumulative incidence of the primary outcome will be estimated using the Kaplan–Meier method. Secondary outcomes involving continuous variables, such as hs-CRP measured at 1, 6, and 12 months, will be analyzed using repeated-measures ANOVA or the Friedman test, as appropriate. For comparisons between two time points, paired t-tests or Wilcoxon signed-rank tests will be applied based on data distribution. Repeated categorical variables will be analyzed using McNemar's test or Cochran's *Q* test.

For the predefined subgroup analysis comparing patients with and without high residual inflammation (hs-CRP ≥ 2 mg/L vs. <2 mg/L at 1 month), continuous variables will be compared using independent samples t-tests or the Mann–Whitney *U* test, and categorical variables using the chi-square test or Fisher's exact test. Kaplan–Meier curves and the log-rank test will be used for time-to-event comparisons, and Cox proportional hazards models will estimate hazard ratios with 95% confidence intervals. To identify predictors of high residual inflammation despite colchicine therapy, logistic regression analysis will be performed using baseline clinical, procedural, and laboratory variables. Both univariable and multivariable models will be constructed.

A pre-specified analysis will compare patient-level data from the MACT II cohort with matched populations from the TICO trial who received either 12-month DAPT or 3-month DAPT followed by P2Y12 inhibitor monotherapy. This comparison is exploratory and intended for contextual interpretation rather than formal hypothesis testing. Propensity score matching will be used to balance baseline characteristics, and time-to-event outcomes will be analyzed using the same methods described above. Sensitivity analyses will include multivariable Cox regression and inverse probability weighting to adjust for potential confounders. While exploratory, this comparative analysis will provide important clinical context and generate hypotheses regarding the potential benefits or limitations of the aspirin-free, inflammation-guided strategy evaluated in MACT II, thereby informing the design and rationale of future randomized trials.

All analyses will be performed using SAS version 9.4 (SAS Institute, Cary, NC, USA). A two-sided *p*-value <0.05 will be considered statistically significant.

### Predefined subgroup of patients and ancillary studies

A predefined subgroup analysis will compare clinical outcomes between patients with high residual inflammation (hs-CRP ≥2 mg/L at 1 month) and those without (hs-CRP <2 mg/L), to assess whether persistent inflammation after PCI is associated with an increased risk of adverse events despite colchicine therapy. In addition, clinical, procedural, and laboratory characteristics will be compared between the two groups to identify factors associated with an inadequate inflammatory response to colchicine.

Pre-specified patient-level comparisons will be conducted between the MACT II cohort and matched populations from the TICO trial who received either 12-month DAPT or 3-month DAPT followed by P2Y12 inhibitor monotherapy. This analysis aims to explore the relative efficacy and safety of colchicine-based therapy in the context of conventional antiplatelet strategies.

### Status

As of the time of manuscript submission, a total of 83 patients have been enrolled in the MACT II trial. Patient recruitment and follow-up are ongoing at participating centers.

## Discussion

The MACT II trial was designed to evaluate the efficacy and safety of an aspirin-free strategy combining ticagrelor monotherapy with low-dose colchicine in patients with ACS undergoing PCI. Building upon the findings of the MACT pilot study (11), which demonstrated the feasibility of colchicine use following early aspirin discontinuation, MACT II incorporates an hs-CRP-guided approach to personalize treatment duration. Patients with hs-CRP ≥2 mg/L at 1 month continue colchicine, while those with hs-CRP <2 mg/L discontinue therapy, allowing for inflammation-guided risk stratification. This single-arm, prospective, multicenter study aims to assess whether this strategy can effectively reduce both residual inflammatory risk and bleeding, while maintaining ischemic protection in a contemporary ACS population.

Early aspirin discontinuation after PCI has been shown to reduce major bleeding without increasing ischemic risk, as demonstrated in trials such as TWILIGHT and TICO (3, 4). These findings support the use of P2Y12 inhibitor monotherapy in selected patients, particularly those at high bleeding risk (1, 2). However, in the acute phase of ACS, heightened inflammation amplifies thrombogenicity, raising concerns about premature aspirin withdrawal. Studies using clopidogrel or low-dose prasugrel monotherapy—such as STOPDAPT-2 ACS and STOPDAPT-3 and—reported increased ischemic events (19, 20), underscoring the need for potent platelet inhibition during this vulnerable period. MACT II addresses this by combining early aspirin discontinuation with standard-dose ticagrelor, initiated immediately after PCI. This strategy aims to minimize bleeding while maintaining sufficient antithrombotic protection in the pro-inflammatory setting of ACS. The addition of colchicine may further counteract inflammation-driven thrombosis during this high-risk window (21).

Residual inflammatory risk after PCI is increasingly recognized as a key determinant of adverse outcomes in patients with ACS, even in the setting of effective antiplatelet therapy. Early anti-inflammatory intervention may therefore be essential in mitigating this risk during the vulnerable post-PCI phase. In the MACT pilot study, colchicine initiated shortly after PCI significantly reduced hs-CRP levels—from 6.1 mg/L (IQR: 2.6–15.9) at 24 h to 0.6 mg/L (IQR: 0.4–1.2) at 1 month (*P* < 0.001) (11). The proportion of patients with high residual inflammation (hs-CRP ≥ 2 mg/L) also declined from 81.8% to 11.8% (11). Comparative data further suggested that colchicine suppressed inflammation more effectively than aspirin (22). MACT II builds on these findings by applying an hs-CRP-guided approach to tailor colchicine duration—limiting therapy in patients with adequate early response while extending it in those with persistent inflammation. This strategy aims to optimize anti-inflammatory benefit, reduce unnecessary drug exposure, and minimize side effects such as diarrhea. As most patients are expected to discontinue colchicine at 1 month, the trial will also assess longitudinal hs-CRP trends to evaluate the durability of inflammatory control after early withdrawal.

Recent trials investigating colchicine in ACS have yielded inconsistent results. The CLEAR SYNERGY (OASIS 9) trial, a large-scale, placebo-controlled study enrolling over 4,000 patients with STEMI, did not demonstrate a reduction in ischemic events and reported a higher rate of non-cardiovascular death in the colchicine group (23). Although CRP levels were significantly reduced at 3 months (2.98 ± 0.19 mg/L in the colchicine group vs. 4.27 ± 0.19 mg/L in the placebo group) (23), the residual inflammatory burden remained substantial. One possible contributing factor was the conduct of the trial during the COVID-19 pandemic, which may have increased baseline and follow-up systemic inflammation through subclinical infection or immune activation (24, 25). Furthermore, colchicine dosing was fixed throughout the study and not adapted based on individual inflammatory response, limiting the ability to target residual inflammatory risk more precisely. The persistently high residual inflammation despite treatment may, at least in part, explain the neutral clinical effect observed in the trial.

In contrast, the MACT II trial, launched before the publication of CLEAR SYNERGY, was independently designed to address residual inflammatory risk through a personalized approach. Colchicine duration is guided by hs-CRP levels at 1 month, allowing therapy to be extended only in patients with persistent inflammation while minimizing unnecessary treatment in those with early resolution. However, similar to CLEAR SYNERGY, colchicine dosing in MACT II remains fixed and is not adjusted according to the magnitude of residual inflammation. Given the persistently elevated CRP levels observed in a subset of patients despite therapy in prior studies, the appropriateness of a one-size-fits-all dosing strategy warrants further consideration. Notably, MACT II incorporates serial hs-CRP assessments, allowing longitudinal evaluation of inflammatory trends even among patients who fail to achieve an early response. These data may provide insight into the dynamics of residual inflammation. Future studies might explore dose adjustments or combination anti-inflammatory strategies to manage persistent inflammation more effectively and further enhance clinical outcomes.

This study has key limitations. First, MACT II is a single-arm, open-label trial without a randomized control group, limiting causal interpretation and safety comparisons with standard care. Planned patient-level comparisons with the TICO trial are exploratory and subject to confounding (4). Second, hs-CRP is measured while patients are still on colchicine, so early responders with low hs-CRP may have treatment withdrawn despite potential benefit; hs-CRP will also be measured at 6 and 12 months to assess longitudinal inflammatory response according to colchicine use. Third, comparisons between patients with and without ongoing inflammation are non-randomized, and outcome differences may reflect baseline risk rather than treatment effect. Fourth, hs-CRP alone may not fully capture vascular or plaque-specific inflammation; additional biomarkers could enhance interpretability. Fifth, the fixed colchicine dose (0.6 mg daily) does not account for bodyweight-related pharmacokinetic variability, which may affect exposure and tolerability. Finally, the use of a single stent platform with ticagrelor may limit generalizability.

The MACT II trial is a prospective, multicenter study designed to evaluate the safety and efficacy of an aspirin-free, inflammation-guided strategy combining ticagrelor monotherapy with low-dose colchicine in patients with ACS undergoing PCI. Early discontinuation of aspirin is expected to reduce bleeding risk, while substituting colchicine may offer additional protection against ischemic events through suppression of residual inflammation. By tailoring colchicine duration according to hs-CRP levels, the study aims to maximize clinical benefit while minimizing unnecessary drug exposure. Through early initiation, individualized treatment, and structured longitudinal biomarker assessment, MACT II seeks to address residual inflammatory risk during the vulnerable post-PCI phase. Although promising, the findings will require confirmation in randomized controlled trials with diverse patient populations to validate this precision inflammation modulation approach in contemporary ACS management.
